# Inverting angiogenesis with interstitial flow and chemokine matrix-binding affinity

**DOI:** 10.1038/s41598-022-08186-0

**Published:** 2022-03-10

**Authors:** Adrian Moure, Guillermo Vilanova, Hector Gomez

**Affiliations:** 1 School of Mechanical Engineering, Purdue University, West Lafayette, IN USA; 2 Department of Mechanical and Civil Engineering, California Institute of Technology, Pasadena, CA USA; 3 LaCàN, Universitat Politècnica de Catalunya-BarcelonaTech, Barcelona, Spain; 4 Weldon School of Biomedical Engineering, Purdue University, West Lafayette, IN USA; 5 Purdue Center for Cancer Research, Purdue University, West Lafayette, IN USA

**Keywords:** Biomedical engineering, Computational biophysics, Computational models

## Abstract

The molecular signaling pathways that orchestrate angiogenesis have been widely studied, but the role of biophysical cues has received less attention. Interstitial flow is unavoidable in vivo, and has been shown to dramatically change the neovascular patterns, but the mechanisms by which flow regulates angiogenesis remain poorly understood. Here, we study the complex interactions between interstitial flow and the affinity for matrix binding of different chemokine isoforms. Using a computational model, we find that changing the matrix affinity of the chemokine isoform can invert the effect of interstitial flow on angiogenesis—from preferential growth in the direction of the flow when the chemokine is initially matrix-bound to preferential flow against the flow when it is unbound. Although fluid forces signal endothelial cells directly, our data suggests a mechanism for the inversion based on biotransport arguments only, and offers a potential explanation for experimental results in which interstitial flow produced preferential vessel growth with and against the flow. Our results point to a particularly intricate effect of interstitial flow on angiogenesis in the tumor microenvironment, where the vessel network geometry and the interstitial flow patterns are complex.

## Introduction

Angiogenesis —the growth of new blood vessels from pre-existing ones— plays a crucial role in cancer metastasis^1^, wound healing^2^, and tissue growth^3^. A better understanding and control of angiogenesis would accelerate progress in oncology^4^, tissue engineering^5^, and regenerative medicine^6^. Angiogenesis^7^ is a multistep process that involves the activation of tip endothelial cells (TECs), the initiation of sprouts, and the growth of new capillaries guided by the motion of tip cells. These steps are controlled by different signaling pathways whose main components are angiogenic growth factors that act as chemokines for tip cells^8^.

**Figure 1:**
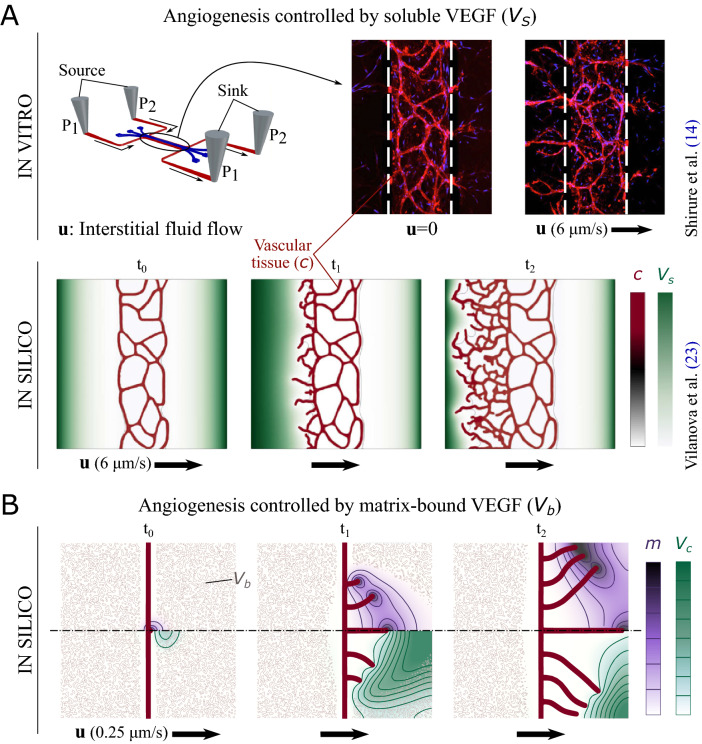
Flow-mediated angiogenesis controlled by (**A**) unbound and (**B**) matrix-bound isoforms of vascular endothelial growth factors (VEGF). Black arrows represent the interstitial flow ${\varvec{u}}$. **(A)** In vitro^14^ and in silico^23^ investigation showed that interstitial flow changes the spatial distribution of unbound (or soluble) VEGF via convective transport. Gradients of soluble VEGF ${ (V_s)}$ are steeper in the opposite direction to the interstitial flow, which induces preferential vascular growth against the flow. Adapted from Shirure et al.^14^ and Vilanova et al.^23^. **(B)** When VEGF is initially bound to the matrix, the proteolytic activity of matrix metalloproteinases (MMPs, secreted by tip cells) is required to cleave the matrix-bound growth factors, which transform into soluble isoforms denoted as cleaved VEGF. Our model shows that the combined transport of MMPs (*m*, top half) and cleaved VEGF ($V_c$, bottom half) produces positive gradients of cleaved VEGF in the direction of the flow near the tip cells, which induce preferential capillary growth with the flow. Top and bottom halves of the plot correspond to the same simulation.

While the biochemical signaling pathways that regulate angiogenesis have been widely studied^8,9^, the biophysical mechanisms have received less attention. Among the latter, fluid flow stands out as a key regulator. Past efforts to understand the impact of flow on angiogenesis have primarily focused on intravascular flow^10^. However, interstitial flow^11^ represents an important biophysical cue that is attracting increasing interest. Recent advances in microfluidics^12^ have allowed to study angiogenesis with controlled interstitial flow^13,14^. The experiments show that interstitial flow produces a dramatic change in the neovasculature. However, the mechanisms by which interstitial flow regulates angiogenesis remain unclear. Some argue that tip cells respond actively to the forces produced by interstitial flow by changing their migration speed and direction^15–18^. Others have argued that interstitial flow affects angiogenesis primarily by modifying the strength and orientation of growth factor gradients without significant changes in the tip cells response to chemokines^14^. What is puzzling is that some experiments reported capillary morphogenesis biased in the direction of the flow^19,20^, others observed vascular growth independent of the flow direction^17^, and, more recently, microfluidic experiments^14,21,22^ and our own simulations^23^ evidenced capillary growth primarily against the flow; see Fig. 1A.

Despite much effort in understanding the impact of interstitial flow on angiogenesis, few have studied the complex interactions between flow and the affinity for matrix binding of different chemokine isoforms. Here, we address this question by comparing how interstitial flow affects angiogenesis in two different scenarios: when the chemokine is initially bound to the matrix and when it is unbound and can be considered a soluble protein. Using a computational model, we find that an adequate combination of flow and chemokine isoform can invert angiogenesis —from growing against the flow for unbound chemokines to growing with the flow for matrix-bound growth factors; see Fig. 1B. Our model indicates that the inversion can be explained using transport arguments only, without resorting to cell active sensing mechanisms. More specifically, the inversion is a result of the different effect of fluid flow on the transport of bound and unbound chemokines. Interestingly, our proposed mechanism of flow-controlled angiogenesis may reconcile experiments that reported capillary growth against and with the flow. While the former^14^ used VEGF$_{121}$, which has very low affinity for fibrin and can be considered soluble; the latter^19^ employed a fibrin-bound VEGF variant that is released proteolytically. We believe that our findings offer a simple and plausible explanation for the controversial role of interstitial flow on angiogenesis. Our results may also help identify failure mechanisms of anti-angiogenic drugs that depend on interstitial flows^24^.

## Results

### The vascular network preferentially grows with the flow when angiogenesis is controlled by matrix-bound chemokines

**Figure 2:**
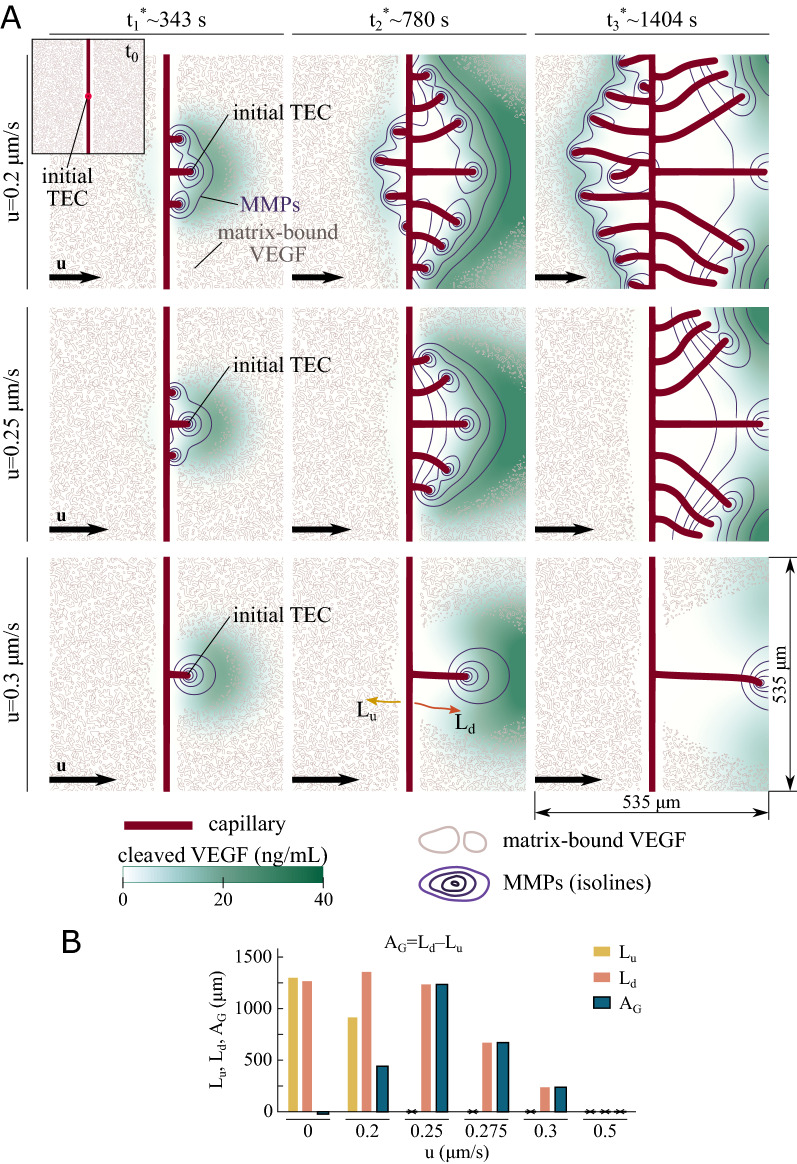
Flow-mediated angiogenesis controlled by matrix-bound chemokines. **(A)** Time evolution of the vascular network (red), cleaved-VEGF distribution (green), MMPs isolines (purple), and contour lines of the matrix-bound VEGF clusters (gray) for interstitial flow $u=0.2$ (top row), 0.25 (center row), and ${0.3}\,{\upmu }\hbox {m s}^{-1}$ (bottom row). The flow is uniform from left to right. The top-left inset shows the initial configuration. Note that the plots identified as $t_i^*$ (where $i=1$, 2, or 3) do not correspond to the same time, but similar times. **(B)** *Asymmetric growth* $(A_G)$ as a function of *u*. $L_u$ and $L_d$ are the total length of the neovasculature growing upstream and downstream, respectively, at $t_3^*$.

We computationally investigate the role of interstitial flow in angiogenesis when the chemokine is initially bound to the matrix; a discussion on flow-mediated angiogenesis when the chemokine is unbound may be found in Vilanova et al.^23^ (see Fig. 1A). The mathematical model and problem configuration used in our simulations is described in Section *Computational Methods*. We suppose that the motion of TECs is guided by gradients of unbound chemokines only. In addition, we assume that the chemokines guiding capillary growth are vascular endothelial growth factors (VEGF). We consider a left-to-right uniform flow with velocity ${\varvec{u}}=(u,0)$. To study the influence of interstitial flow, we run simulations with different velocities *u*. For each velocity, we run several simulations with different initial distributions of matrix-bound VEGF (see Fig. 2A) to show that the random location of the matrix-bound VEGF clusters does not affect the results. In Fig. 2A we plotted the simulation results for $u=0.2$, 0.25, and ${0.3}\,{\upmu }\hbox {m s}^{-1}$, from the top to the bottom row, respectively. Each row displays the vascular network (red) and the cleaved-VEGF distribution (green) at three different times. The plots also show the contour lines of the matrix-bound VEGF clusters (gray) and the MMPs isolines (purple). The three plots identified as the time $t_i^*$ (where $i=1$, 2, or 3) do not correspond to the same time, but to close times. We have not plotted the same time for the three simulations because we used an adaptive time stepping solver. Figure 2A shows that capillaries grow more prominently in the direction of the flow. Independent of the flow velocity, the simulations picture a common behavior: The initial tip cell secretes MMPs, which experience diffusion and convection. Convective transport provokes the MMPs concentration to be higher downstream the parent vessel, which induces higher cleavage rates of matrix-bound VEGF. The cleaved-VEGF concentration is higher downstream the parent vessel (see $t_1^*$ in Fig. 2A), which induces TEC activation on the downstream side of the parent capillary. To achieve the activation of new TECs, the upstream diffusive transport of cleaved VEGF must prevail over convective transport, so that enough cleaved VEGF reaches the parent vessel. While low fluid velocities induce TEC activation on both sides of the parent vessel (see $u={0.2}\,{\upmu }\hbox {m s}^{-1}$ in Fig. 2A), intermediate velocities allow TEC activation on the downstream side of the parent vessel only (see $u={0.25}\,{\upmu }\hbox {m s}^{-1}$ in Fig. 2A). In the case of low velocities, the activation of the upstream tip cells occurs after the activation of the downstream ones. Thus, the capillary network downstream the parent vessel is more developed than the upstream network; see $u={0.2}\,{\upmu }\hbox {m s}^{-1}$ in Fig. 2A. Velocities higher than $u={0.5}\,{\upmu }\hbox {m s}^{-1}$ wash away MMPs and cleaved VEGF, which prevents the growth of new capillaries (see Fig. 2B).

### The effectiveness of vascular growth with the flow is maximum at intermediate velocities

The simulation results in Fig. 2A show that interstitial flow affects capillary growth in two ways when angiogenesis is controlled by matrix-bound chemokines. On the one hand, interstitial flow introduces asymmetry such that new vessels grow more prominently in the direction of the flow. On the other hand, as the fluid velocity increases, the density of the vascular network decreases. These two effects suggest the idea of quantifying the effectiveness of vascular growth with the flow. To do that, we define the *asymmetric growth* as $A_G=L_d-L_u$, where $L_d$ and $L_u$ are the total length of the new vessels growing downstream and upstream the parent vessel, respectively, at the final stage of the simulation. We plotted $A_G$, $L_d$, and $L_u$ for different interstitial fluid velocities in Fig. 2B. In the no-flow scenario, $A_G$ is nearly zero due to the non-preferential direction of capillary growth (results shown in Fig. 4A). As the fluid velocity increases, $A_G$ increases because $L_u$ decreases while $L_d$ maintains a high value; compare $u={0.2}\,{\upmu }\hbox {m s}^{-1}$ and $u={0.25}\,{\upmu }\hbox {m s}^{-1}$ in Fig. 2. After a certain velocity, $A_G$ begins to decrease because $L_u$ has vanished and $L_d$ starts to decrease; compare $u={0.25}\,{\upmu }\hbox {m s}^{-1}$ and $u={0.3}\,{\upmu }\hbox {m s}^{-1}$ in Fig. 2. For high velocities, $A_G$ is zero because both $L_u$ and $L_d$ are zero. $A_G$ is maximum at the intermediate velocity $u_A$ and becomes zero at the *limit* velocity $u_L$. In our simulations, $u_A\approx {0.25}\,{\upmu }\hbox {m s}^{-1}$ and $u_L\approx {0.5}\,{\upmu }\hbox {m s}^{-1}$. The impact of the MMPs secretion rate and the matrix-bound VEGF initial distribution on $A_G$ is explained in the Supplementary Information.

### The pre-existing vascular pattern affects the growth of the new vessels

**Figure 3:**
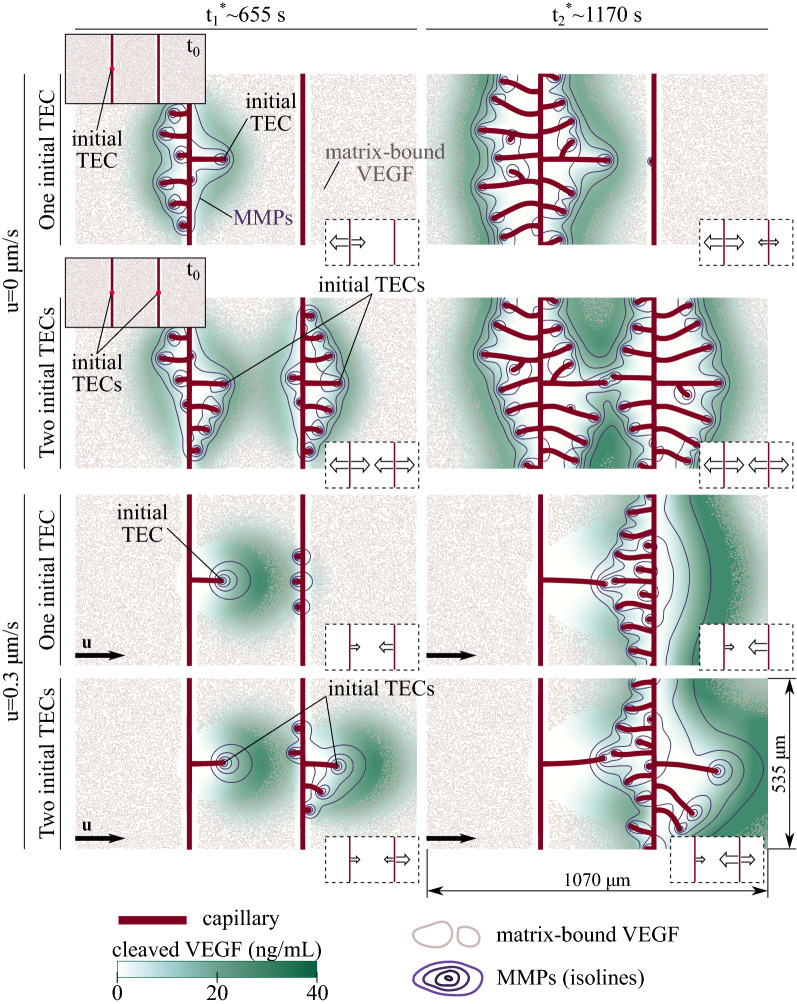
Angiogenesis controlled by matrix-bound chemokines in a two-parent vessel configuration. No-flow (upper half) and flow-mediated ($u={0.3}\,{\upmu }\hbox {m s}^{-1}$, bottom half) angiogenesis for initial conditions with one tip cell in the left parent vessel (first and third rows) and tip cells in both parent vessels (second and fourth rows). The two solid-line insets show the two initial configurations. Capillary network (red), cleaved-VEGF distribution (green), MMPs isolines (purple), and matrix-bound VEGF contour lines (gray) at two different times. Note the time $t_i^*$ (where $i=1$ or 2) does not represent the same time for the four simulations, but similar times. The dashed-line inset at the bottom-right corner of each panel indicates the direction and degree of vascular growth ( averaged over several simulations, the size of the arrows is proportional to the number of growing vessels).

We move to a more complex scenario in which we study angiogenesis in an environment with two parent vessels. We resort to a computational domain twice the size in the horizontal direction of the previous examples (more details about the problem configuration may be found in Supplementary Information, *Initial Conditions*). We study two cases. In the first case, we assume there is one initial active tip cell in the left parent vessel; see Fig. 3, top-row inset. In the second case, both the right and left parent vessels contain an initial TEC; see Fig. 3, second-row inset. For both cases, we run no-flow simulations and simulations with fluid velocity $u={0.2}\,{\upmu }\hbox {m s}^{-1}$ and $u={0.3}\,{\upmu }\hbox {m s}^{-1}$. Figure 3 displays the results corresponding to $u=0$ and $u={0.3}\,{\upmu }\hbox {m s}^{-1}$. The simulation results for $u={0.2}\,{\upmu }\hbox {m s}^{-1}$ may be found in Supplementary Fig. S2.

In the no-flow scenario, new vessels do not exhibit a preferential direction of growth with respect to the parent capillaries. In the case of one initial tip cell, at the early stages, new sprouts emerge only from the left parent vessel; see Fig. 3, top row. No sprouts emerge from the right parent vessel until late stages of the simulation, when the neovasculature of the left parent vessel approaches the right parent vessel. At that point, the cleaved VEGF released by the new capillaries reaches the right parent vessel and triggers capillary growth; see $t_2^*$ in Fig 3, top row. The right parent vessel displays a non-preferential direction of vascular growth with respect to the parent vessel (results not shown). When both parent vessels possess an initial TEC (see Fig 3, second row), new capillaries emerge simultaneously from both parent vessels without a preferential direction of growth.

The simulation results of flow-mediated angiogenesis show a more interesting scenario. In the one-initial-TEC case, the upstream (i.e., the left) parent vessel displays angiogenesis with the flow; see Fig. 3, third row. Interstitial flow transports the cleaved VEGF released by the initial TEC to the vicinity of the downstream parent vessel. Since the transformation of matrix-bound into cleaved VEGF does not occur near the downstream parent vessel, that vessel perceives cleaved VEGF as an unbound chemokine secreted by an external source. That is the case we studied in Vilanova et al.^23^ (see Fig. 1A), in which the capillaries grow more prominently against the flow. For this reason, the neovasculature of the downstream parent vessel grows against the flow; see Fig. 3, third row. The case with two initial TECs is even more interesting; see Fig. 3, bottom row. At the initial times, the new capillaries grow with the flow in both parent vessels. As the vasculature grows, the cleaved VEGF released by the upstream tip cells is transported by the flow, reaches the downstream parent vessel, and triggers the growth of new capillaries against the flow. While the upstream parent vessel displays angiogenesis with the flow, the downstream parent vessel undergoes angiogenesis simultaneously with and against the flow.

These results suggest that the geometry of the pre-existing vascular network plays an important role in flow-mediated angiogenesis driven by matrix-bound chemokines. The pre-existing vascular pattern may determine whether the new vessels grow with or against the flow. The results also suggest that the presence of both unbound and matrix-bound chemokines in the vascular environment provokes a competition between angiogenesis with and against the flow. We run the same set of simulations with different distances between the parent vessels. The results show that, as the parent vessels separation increases, the new sprouts growing against the flow in the downstream parent vessel emerge later (see Supplementary Fig. S3). The influence of the MMPs secretion rate and the matrix-bound initial distribution in the two-parent-vessel configuration is explained in the Supplementary Information. These results illustrate a mechanism to alter the preferential direction of vascular growth based on the pre-existing vascular geometry.

## Discussion

We showed in Vilanova et al.^23^ that capillary growth is more prominent against the flow when angiogenesis is orchestrated by a soluble isoform of VEGF; see Fig. 1A. There, we compared our model predictions with an in vitro experiment that solely considered soluble VEGF. However, under physiological conditions, chemokines may be present in unbound and matrix-bound isoforms^25^. Indeed, matrix-bound is the dominant VEGF isoform in one of the experiments that reported capillary morphogenesis with the flow^19^. Here, we couple the dynamics of unbound and matrix-bound VEGF into our earlier angiogenesis model^26^. We also include the dynamics of MMPs, which provoke the proteolytic cleavage of matrix-bound VEGF. Our model successfully reproduces capillary growth controlled by matrix-bound chemokines and allows us to study flow-mediated angiogenesis when matrix-bound is the dominant chemokine isoform. The model results show that interstitial flow can invert angiogenesis from growing against the flow when the chemokine is initially unbound, to growing with the flow when the chemokine is bound to the matrix. Our results suggest that the asymmetric growth in the direction of the flow is more prominent at intermediate fluid velocities. The results also show that the pre-existing vascular pattern may affect capillary growth. Once matrix-bound VEGF transforms into cleaved VEGF and is transported far from the region where VEGF is released, the cleaved isoform plays the role of soluble VEGF produced by an external source in Vilanova et al.^23^, i.e., induces capillary growth against the flow. Thus, in the presence of both unbound and matrix-bound chemokines, the effect of interstitial flow on vascular growth is controlled by the local balance between the chemokine isoforms.

One of the key assumptions of our model is that interstitial flow affects angiogenesis by modifying the spatial distributions and gradients of soluble proteins. While this assumption neglects mechanotransduction effects and may be seen as a model limitation, it does illustrate that transport effects alone can offer a potential explanation of the experimentally-observed differences^14,17,19,21,22^ in flow-mediated angiogenesis: Vascular growth is more prominent with the flow when chemokines are initially bound to the matrix, while capillaries preferentially grow against the flow when chemokines are unbound; compare Fig. 1A and B. This assumption also allows us to avoid the challenging task of modeling cell sensing mechanisms such as flow-enhanced TEC activation or preferential TEC motion along flow-induced pressure gradients^27^.

Few experiments reported capillary growth in the direction of the flow. Those experiments resort to different experimental setups such as, e.g., single endothelial cells in fibrin gel matrices^19^ and a microfluidic device in which the vascular tissue is located in two parallel channels at the edges of the device^17^. None of these configurations correspond exactly to the simulations analyzed here (see Figs. 2A and 3). In addition, the matrix-bound VEGF distribution in the experiments is not evident. For these reasons, we are unable to quantitatively compare our results of angiogenesis controlled by matrix-bound chemokines with experiments. One of the reasons for the lack of experiments is the number of challenges that must be overcome to conduct experiments in flow- and chemokine-controlled conditions due to the strong coupling between the vascular network, the interstitial flow, and the distribution of the different chemokine isoforms^14,22^. The flow modifies the spatial distribution of soluble chemokines, whereas the growth of new capillaries alters the flow^23^ and promotes the release of matrix-bound chemokines. Our model may help to design new experimental setups that shed more light into (i) the role played by interstitial fluid in angiogenesis and (ii) the competition between matrix-bound and unbound chemokines. For instance, the two-parent-vessel configuration shown in Fig. 3 could be used in an experiment to study how the parent vessels separation controls the time scale for the activation of preferential growth against the flow. This experiment could shed light into the effect of interstitial flow on tumor-induced angiogenesis where the vascular networks have abnormal inter-capillary distances.

## Computational methods

### Mathematical model

Previous models that investigate angiogenesis controlled by different chemokine isoforms focus on the transformations between the different isoforms^19,28–30^. Those models analyze the chemokine spatial distribution created by a single TEC fixed in space. Only Milde et al.^31^ accounts for the motion of the tip cells to reproduce capillary growth. However, Milde et al.^31^ neglects convective transport. In fact, all those works neglect the effect of interstitial flow except Helm et al.^19^. Compared to those models, we consider both convective transport and the growth of the vascular network, which allows us to study the influence of interstitial flow in a growing vascular network.

We leverage a computational model of angiogenesis^26,32^ that qualitatively reproduces experiments of capillary growth driven by vascular endothelial growth factor (VEGF), one of the most potent pro-angiogenic factors^25,33,34^. The model is based on the premise that an abnormal increase in the VEGF concentration near the vessel wall induces changes in the phenotype of the endothelial cells, such that one cell (or a group of cells) becomes migratory (i.e., the TECs) while the surrounding cells remain quiescent or become proliferative (i.e., the stalk endothelial cells). Although there is evidence that tip cells sense fluid forces and react to them^14^, the model assumes that tip cells migrate using chemotactic guidance only, i.e., simply following gradients of VEGF. The impact of flow on angiogenesis emerges from the change of VEGF gradients produced by the flow.

**Figure 4:**
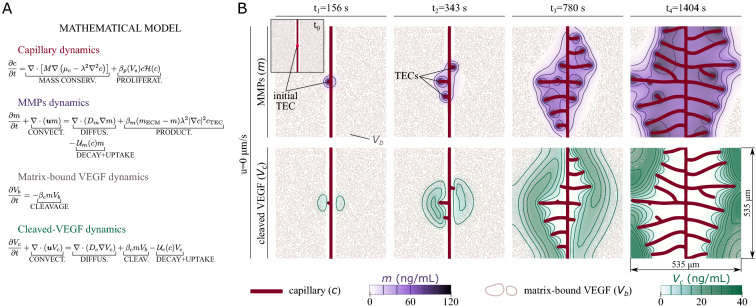
Computational modeling and simulation of flow-mediated angiogenesis driven by matrix-bound chemokines. **(A)** Model equations. Tip cells are modeled as discrete agents and follow the set of rules described in Supplementary Information, *Discrete compartment*. **(B)** Problem configuration used in the paper. The results correspond to capillary growth controlled by matrix-bound VEGF in the absence of interstitial flow. The top-left inset shows the initial configuration, which includes the matrix-bound VEGF clusters, a parent vessel, and a non-motile TEC secreting MMPs. The initial TEC begins motion once it fulfills all the conditions for TEC activation. The main panels show the MMPs (top row) and cleaved-VEGF (bottom row) distributions at four different times. Both rows display the capillary network (red) and the contour lines of the matrix-bound VEGF clusters (gray).

To be able to study the impact of different chemokine isoforms, we extend our earlier model^26^ which was designed to simulate angiogenesis driven by soluble isoforms of VEGF. The extended model (see Fig. 4A) includes a bound $(V_b)$ and unbound (cleaved, $V_c$) isoform of VEGF, as well as the dynamics of MMPs (*m*). The proteolytic activity of MMPs provokes the release of matrix-bound VEGF^35,36^, which transforms into cleaved VEGF. Whereas matrix-bound VEGF is attached to the matrix fibers, both MMPs and cleaved VEGF may be transported by the interstitial flow ${\varvec{u}}$. The MMPs, matrix-bound VEGF, and cleaved-VEGF dynamics are governed by convection-diffusion equations that also account for the reactions between these components; see Fig. 4A. We assume that MMPs are secreted by tip cells and both MMPs and cleaved VEGF undergo degradation and uptake by the endothelial cells. We use the phase field *c* to capture the growth of the capillary network. The phase-field representation^37,38^ allows us to simulate time-evolving vascular patterns in a fixed computational domain. The capillary dynamics is controlled by a Cahn-Hilliard equation extended with an endothelial cell proliferation term; see Fig. 4A. The model includes a discrete compartment that accounts for the activation and motion of TECs, which are modeled as discrete agents. More details about the model and the discrete agents may be found in Supplementary Information, *Mathematical model*.

### Numerical simulation

We consider a square computational domain of size $535\times {535}\,{\upmu }\hbox {m}^{2}$ with an initial (or parent) vertical vessel at the center of the domain. The domain contains 3200 circular matrix-bound VEGF clusters randomly distributed and no closer than ${20}\,{\upmu }\hbox {m}$ to the initial vessel. We assume the domain does not contain MMPs or cleaved VEGF at the initial time. Thus, soluble VEGF in our simulations is produced from the cleavage of matrix-bound VEGF only. For the simulations shown in Fig. 3, we consider a rectangular domain of $1070\times {535}\,{\upmu }\hbox {m}^{2}$ with 6400 matrix-bound VEGF clusters. The initial configuration cannot induce TEC activation due to the lack of cleaved VEGF. To trigger angiogenesis, we place a non-motile TEC secreting MMPs at the center of the parent vessel. The non-motile TEC becomes motile when it satisfies the conditions for TEC activation. Another alternative would be to include an initial amount of cleaved VEGF in the domain. In case of flow-mediated angiogenesis, the latter option would introduce an initial asymmetry caused by convective transport of cleaved VEGF prior to the activation of the first TEC. In addition, the initial distribution of cleaved VEGF might affect the growth of the neovasculature. To avoid predetermined asymmetries, we consider initial non-motile TECs in all the simulations shown in this paper. We assume free-flux boundary conditions. More details about the initial conditions, parameter values, and numerical implementation may be found in the Supplementary Information.

Before studying the effect of flow, we test our model in an environment without flow. We run several simulations with different initial distributions of matrix-bound VEGF to verify that the overall behavior of the vascular network does not depend on the initial distribution of matrix-bound VEGF. We show the results of one of those simulations in Fig. 4B. The figure shows the vascular network (red), the contour lines of the matrix-bound VEGF clusters (gray), the MMPs distribution (purple, top row), and the cleaved-VEGF distribution (green, bottom row) at four different times. Figure 4B captures the main features of the model: The initial tip cell secretes MMPs, which diffuse and release matrix-bound VEGF from the matrix fibers. Once the cleaved-VEGF concentration at the initial TEC reaches the activation threshold, the TEC begins to move following the gradient of cleaved VEGF; see $t_1$ in Fig. 4B. As the initial TEC moves, MMPs spread over a larger area and more cleaved VEGF is released. The *lateral inhibition mechanism*^39^ impedes new tip cells from emerging in the immediate vicinity of existing ones. New sprouts grow left or right (see $t_2$ in Fig. 4B) depending on the spatial distribution of cleaved VEGF, which displays mild perturbations caused by the random distribution of the matrix-bound VEGF clusters. Behind the tip cells, the stalk cells proliferate and promote the elongation of the new capillaries^40,41^. The results show that matrix-bound VEGF completely transforms into cleaved VEGF in the surroundings of the capillaries. Our model results do not display a preferential direction of capillary growth with respect to the parent vessel in an environment without flow.

## Supplementary Information

Supplementary Information.

## Data Availability

The data supporting the findings of this study are available from the corresponding author upon reasonable request.

## Code availability

The code used in this study is available from the corresponding author upon reasonable request.
