# Supplementary material for: Inverting angiogenesis with interstitial flow and chemokine matrix-binding affinity
Source: Sci Rep. 2022 Mar 10;12:4237. doi: 10.1038/s41598-022-08186-0 (PMC8913640; doi:10.1038/s41598-022-08186-0)
Supplement: Supplementary file 1 — Supplementary Information. [file 41598_2022_8186_MOESM1_ESM.pdf]

Supplementary Information

Inverting angiogenesis with interstitial flow and chemokine matrix-binding affinity

Adrian Moure[1,2,*], **Guillermo Vilanova**[3], **and Hector Gomez**[1,4,5]

[1]School of Mechanical Engineering, Purdue University, West Lafayette, IN, USA.
[2]Department of Mechanical and Civil Engineering, California Institute of Technology, Pasadena, CA, USA.
[3]LaCàN, Universitat Politècnica de Catalunya-BarcelonaTech, Barcelona, Spain.
[4]Weldon School of Biomedical Engineering, Purdue University, West Lafayette, IN, USA.
[5]Purdue Center for Cancer Research, Purdue University, West Lafayette, IN, USA.
[*]amoure@caltech.edu

Mathematical model

Our mathematical model is an extension of our previous work[1,2], in which we studied angiogenesis controlled by soluble VEGF. Here, we propose a hybrid model for flow-mediated angiogenesis in the presence of different VEGF isoforms. The continuous compartment of the model accounts for the dynamics of the capillaries (c), MMPs (m), matrix-bound VEGF (V_b), and cleaved VEGF (V_c). The discrete compartment controls the activation, motion, and deactivation of tip endothelial cells (TECs), which are treated as discrete agents. In our model, we consider that the interstitial fluid velocity $\boldsymbol{u}$ is given. In Vilanova et al.[3] we used a reduced version of the model to study flow-mediated angiogenesis controlled by soluble VEGF only.

Continuous compartment

We use the phase-field method[4,5] to capture the capillary network. The phase-field method is a technique that permits to reformulate a moving-boundary problem in a known and fixed domain. This allows us to solve problems with moving interfaces using a fixed and regular mesh. In our model, the phase field c takes the value 1 at the capillaries, -1 at the extracellular environment, and smoothly transitions from 1 to -1 at the interface (i.e., the vessel wall). The capillary dynamics is controlled by the equation

$$\frac{\partial c}{\partial t} = \nabla \cdot \left[M \nabla \left(\mu_c - \lambda^2 \nabla^2 c \right) \right] + \beta_p(V_c) c \mathscr{H}(c), \tag{1}$$

which is a Cahn-Hilliard equation[6] extended with a proliferative term. In Eq. (1), M is the mobility which we assume constant, λ is a parameter proportional to the interface width, and $\mu_c = c(c^2-1)$ is the chemical potential, which is derived from a double-well potential with minima at $c=1$ and $c=-1$. The last term in Eq. (1) accounts for the endothelial cell proliferation. $\mathscr{H}(c)$ is a smeared-out Heaviside function and $\beta_p(V_c)$ is a function that limits the endothelial cell proliferation for high cleaved-VEGF concentrations such that

$$\beta_p(V_c) = \begin{cases} \beta V_c & \text{if } V_c < V_{c,p}, \\ \beta V_{c,p} & \text{if } V_c \geq V_{c,p}. \end{cases} \tag{2}$$

Here, the parameters β and $V_{c,p}$ are the proliferation rate and the limiting cleaved-VEGF concentration, respectively. We explain below how we couple the phase-field equation with the TECs motion (see Section *Discrete Compartment*).

We assume that MMPs are secreted by tip endothelial cells and can be transported through the extracellular environment via diffusion and convection. MMPs experience a natural degradation and can intravasate the vessel walls. The evolution equation for the MMPs dynamics is

$$\frac{\partial m}{\partial t} + \nabla \cdot (\boldsymbol{u} m) = \nabla \cdot (D_m \nabla m) + \beta_m (m_{\text{ECM}} - m) \lambda^2 |\nabla c|^2 c_{\text{TEC}} - \mathscr{U}_m(c) m, \tag{3}$$

where D_m is the diffusion coefficient, β_m is the MMPs secretion rate, and m_{ECM} is the maximum MMPs concentration. $\lambda^2|\nabla c|^2 c_{\mathrm{TEC}}$ is a non-dimensional term that localizes MMPs production to the TEC's surface. Finally, the function $\mathscr{U}_m(c)$ accounts for the MMPs degradation and uptake by the vessels. The function $\mathscr{U}_m$ is written as

$$\mathscr{U}_m(c) = \begin{cases} -U_{m,d}c & \text{if } c < 0, \\ U_{m,u}c & \text{if } c \geq 0, \end{cases} \tag{4}$$

where $U_{m,d}$ is the MMPs decay rate and $U_{m,u}$ is the uptake rate.

Matrix-bound VEGF is fixed to the matrix fibers. MMPs provoke the proteolytic cleavage of matrix-bound VEGF at a rate proportional to the MMPs and matrix-bound VEGF concentrations. The evolution equation for matrix-bound VEGF can be expressed as

$$\frac{\partial V_b}{\partial t} = -\beta_c m V_b, \tag{5}$$

where β_c is the VEGF cleavage rate.

Matrix-bound VEGF transforms into cleaved VEGF, which can undergo diffusive and convective transport. In addition, cleaved VEGF experiences natural degradation and absorption by endothelial cells. The dynamics of cleaved VEGF is controlled by the equation

$$\frac{\partial V_c}{\partial t} + \nabla \cdot (\boldsymbol{u} V_c) = \nabla \cdot (D_c \nabla V_c) + \beta_c m V_b - \mathscr{U}_c(c) V_c, \tag{6}$$

where D_c is the cleaved-VEGF diffusion coefficient and the function $\mathscr{U}_c(c)$ accounts for the cleaved-VEGF natural decay and the endothelial cell uptake. The function $\mathscr{U}_c$ is expressed as

$$\mathscr{U}_c(c) = \begin{cases} -U_{c,d}c & \text{if } c < 0, \\ U_{c,u}c & \text{if } c \geq 0, \end{cases} \tag{7}$$

where $U_{c,d}$ and $U_{c,u}$ are the cleaved-VEGF decay rate and the endothelial cell uptake rate, respectively. Eq. (6) and the evolution equation of soluble VEGF in Vilanova et al.[3] only exhibit one difference: the VEGF production term (β_c-term). Here, the production of unbound (cleaved) VEGF is strictly restricted to the cleavage of matrix-bound VEGF, whereas in Vilanova et al.[3] unbound (soluble) VEGF production is restricted to external sources.

Discrete compartment

We model TECs as circular discrete agents of radius R_{TEC}. A new TEC can emerge at any point of the domain if the following conditions are achieved: (1) $c > c_{\mathrm{act}}$, (2) $V_c > V_{c,\mathrm{act}}$, (3) $d_{\mathrm{TEC}} > \delta_4$, and (4) $d_c > R_{\mathrm{TEC}}$, where c_{act}, $V_{c,\mathrm{act}}$, and δ_4 are parameters, d_{TEC} is the distance to the closest TEC, and d_c is the distance to the closest vessel wall (i.e., the locus of points $\boldsymbol{x}$ such that $c(\boldsymbol{x}) = 0$). Any spatial point that meets these conditions becomes the center of the TEC. The first condition enforces the TEC to appear inside the capillary. The second condition represents a threshold of cleaved-VEGF concentration for TEC activation. The third condition accounts for the lateral inhibition mechanism[7], which impedes the activation of more than one TEC in the same region. The last condition prevents any part of the TEC from emerging outside the capillary when the TEC becomes active. Once active, the TEC moves with velocity $\boldsymbol{v}_{\mathrm{TEC}} = \chi \frac{\nabla V_c}{|\nabla V_c|}$, where χ is the chemotactic velocity. During TEC migration, TECs develop filopodia that sense the surrounding environment and may modify the direction of migration. We model filopodia by checking an annular sector centered at the TEC's center. The annular sector has an angle of $2\pi/3$ centered around the direction of migration, an internal radius of $2R_{\mathrm{TEC}}$, and an external radius of $4R_{\mathrm{TEC}}$. If $c > 0.9$ at any point in the annular sector, the direction of migration changes and the TEC moves towards that point. TEC deactivation is the loss of its motile condition. TECs deactivate if $V_c < V_{c,\mathrm{act}}$ at the center of the TEC. TECs also deactivate if they encounter another TEC or capillary during its migration (anastomosis), i.e., if $c > 0.9$ at any point distant $1.5R_{\mathrm{TEC}}$ from the TEC's center in a circular arc with angle $2\pi/3$ centered around the direction of migration. After deactivation, the TEC becomes a quiescent endothelial cell which might experience a subsequent TEC activation.

The discrete agents are coupled with the capillary equation; see Eq. (1). We impose the value of the phase field c in the circular region occupied by the TECs such that $c = 1$. By doing that, we consider the TECs are part of the capillary system.

Boundary conditions

We assume free-flux boundary conditions, which may be expressed as

$$M\nabla\left(\mu_c - \lambda^2 \nabla^2 c\right) \cdot \boldsymbol{n} = 0, \tag{8}$$

$$M\lambda^2\nabla^2 c = 0, \quad (9)$$

$$D_m \nabla m \cdot \boldsymbol{n} = 0, \quad (10)$$

$$D_c \nabla V_c \cdot \boldsymbol{n} = 0, \quad (11)$$

where $\boldsymbol{n}$ is the outward normal vector to the domain.

Parameter values

We listed the parameter values used in our simulations in Table 1. The parameter values associated with the discrete agents, the capillary dynamics (see Eq. (1)), and the unbound (cleaved) VEGF dynamics (see Eq. (6)) are explained in Vilanova et al.[3]. The parameters related to the MMPs dynamics (see Eq. (3)) and the cleavage of matrix-bound VEGF (see Eq. (5)) are taken from previous models that studied the reactions between MMPs and the different VEGF isoforms[8,9]. To estimate some of the parameters from those works[8,9], we assume that TECs are circles of radius R_{TEC} and the MMPs molecular mass is 62.5 kDa.

Initial conditions

We consider two types of computational domains: a square of $535 \times 535\,\mu\text{m}^2$ and a rectangle of $1070 \times 535\,\mu\text{m}^2$. In both domains, we consider as initial conditions 12.5 µm width vertical vessels. In the square domain we consider one initial (parent) vessel, whereas in the rectangular domain we consider two initial vessels. The parent vessels separation is $d_{\text{cap}} = 356.66\,\mu\text{m}$. We assume the initial distributions of MMPs and cleaved VEGF are null for both domains. For the initial distribution of matrix-bound VEGF, we consider matrix-bound VEGF is present in the form of circular clusters of radius 3 µm with uniform concentration of $120\,\text{ng}\,\text{mL}^{-1}$. We place 3200 clusters in the square domain and 6400 clusters in the rectangular domain. The clusters are randomly distributed and their location must follow two conditions: (1) the center of the cluster cannot be closer than 20 µm to the parent vessels and (2) the clusters center cannot be closer than 6 µm to the center of any other cluster (i.e., no clusters overlap). Since we consider the initial concentration of cleaved VEGF is null in the entire domain, we trigger angiogenesis by placing an initial TEC (or two TECs in some cases) in the center of the parent vessel. The initial TEC (or TECs) secretes MMPs from $t = 0\,\text{s}$ but cannot move until all the conditions for TEC activation are achieved at the TEC center; see Section *Discrete compartment*. Once fully active, the initial TEC (or TECs) behaves as a regular TEC.

Numerical implementation

We solve the partial differential equations (PDEs) that constitute the continuous compartment by using Isogeometric Analysis (IGA)[10]. IGA is a generalization of the Finite Element Method that uses B-splines and Non-Uniform Rational B-splines (NURBS) as basis functions. The use of B-splines and NURBS permits a straightforward treatment of higher-order PDEs such as Eq. (1). We derive the weak form of the problem by multiplying Eqs. (1, 3, 5, 6) with weighting functions and integrating in space. We then integrate by parts according to the boundary conditions. For the spatial discretization, we substitute the unknowns and the weighting functions by B-splines. We use a uniform mesh composed of 256×256 quadratic $\mathscr{C}^1$-continuous elements in the computational domain of size $535 \times 535\,\mu\text{m}^2$. In the computational domain of size $1070 \times 535\,\mu\text{m}^2$, we double the number of elements in the x-direction. The use of quadratic $\mathscr{C}^1$-continuous B-splines allows us to obtain a well-defined Galerkin form. For the time integration, we use the generalized-α method[11,12]. This method requires the solution of a non-linear system each time step. We use the Newton–Raphson (NR) method to solve the non-linear system. We implement an adaptive time stepping scheme based on the number of NR iterations. The time step size slightly increases (or decreases) if the number of NR iterations in the previous time step is low (or high). At the beginning of each time step, prior to the NR solver, we evaluate the discrete compartment. We check the activation and deactivation of TECs and update their location according to the explanation provided in Section *Discrete compartment*. We next update the phase-field c by overwriting c in the region occupied by the TECs. To do that, we resort to the notion of template functions[1], which produce a 2D generalization of the 1D solution to the Cahn-Hilliard equation. We overwrite the phase-field c with the template of each TEC, which provides a phase field with the required smoothness across the capillary wall (i.e., the interface).

More details about the numerical implementation of the continuous and discrete compartments and their coupling may be found in our previous works[1–3].

Single-parent-vessel configuration: Influence of MMPs secretion rate and matrix-bound VEGF initial distribution

In addition to the simulations shown in the main text, we have also studied the influence of the MMPs secretion rate and the initial distribution of matrix-bound VEGF in the single-parent-vessel configuration. We first analyze the influence of the MMPs secretion rate by running simulations with different MMPs secretion rates (β_m) for no-flow and flow-mediated angiogenesis. We consider the same simulation setup used in the main text (see, e.g., Fig. 4*B* in the main text). In Fig. S1 we plotted the final state of four simulations corresponding to low ($\beta_m = 0.03\,\mathrm{s}^{-1}$, top row) and high ($\beta_m = 1.65\,\mathrm{s}^{-1}$, bottom row) MMPs secretion rates for interstitial flow $u = 0\,\mu\mathrm{m\,s}^{-1}$ (left column) and $u = 0.3\,\mu\mathrm{m\,s}^{-1}$ (right column). Note that we consider a left-to-right uniform flow $\boldsymbol{u} = (u, 0)$. The four snapshots show the capillary network (red), the cleaved-VEGF distribution (green), the MMPs isolines (purple), and the contour lines of the matrix-bound VEGF clusters (gray). In the absence of interstitial flow, neither low or high β_m exhibit a preferential direction of capillary growth with respect to the parent vessel; see Fig. S1, left column. While low values of β_m slow down capillary growth (extremely low values are unable to trigger angiogenesis), high values of β_m do not exhibit a significant difference compared to intermediate values of β_m; compare the bottom-left panel in Fig. S1 with time t_4 in Fig. 4*B* in the main text. When angiogenesis is mediated by interstitial flow, low values of β_m do not induce vessel growth, while high values of β_m enhance angiogenesis compared to intermediate values of β_m; compare the bottom-right panel in Fig. S1 with the bottom-right panel in Fig. 2*A* in the main text.

In the main text we introduce the concept of *asymmetric growth* to measure the effectiveness of angiogenesis with the flow (i.e., flow-mediated angiogenesis controlled by matrix-bound VEGF). We define *asymmetric growth* as $A_G = L_d - L_u$, where L_d and L_u are the total length of the neovasculature growing downstream and upstream the parent vessel, respectively, at the final state of the simulation. The curve $A_G = A_G(u)$ gradually increases from its initial null value, exhibits a maximum ($A_{G,m}$) at the intermediate velocity u_A, and decreases until A_G vanishes at the limit velocity u_L; see Fig. 2*B* in the main text. The simulation results show that, as β_m increases, u_L and u_A increase, while $A_{G,m}$ increases until reaching a plateau. Note that $A_{G,m}$ takes the same value for intermediate and high β_m. This is caused by the *lateral inhibition mechanism*, which limits the number of TECs (and hence, the number of new vessels) in a certain region. In conclusion, higher MMPs secretion rates improve the effectiveness of angiogenesis with the flow.

We have also analyzed the influence of the initial distribution of the matrix-bound VEGF clusters. To do that, we run simulations in which we modify the number of matrix-bound VEGF clusters, the radius of the clusters, and the number and radius of the clusters simultaneously. The results show that the influence of the matrix-bound VEGF initial distribution can be explained through the total amount of matrix-bound VEGF available at the initial time, which we refer to as N_b. The behavior of flow-mediated angiogenesis with respect to N_b is analogous to the behavior with respect to β_m (see the previous paragraphs). In fact, Fig. S1 could represent the final state of simulations in which we modify the initial amount of matrix-bound VEGF (N_b) instead of the MMPs secretion rate (β_m), where N_b is proportional to β_m. For $u = 0\,\mu\mathrm{m\,s}^{-1}$, low values of N_b slow down and even prevent angiogenesis, while the capillary patterns produced by high values of N_b are similar to those produced by intermediate N_b values. In case of flow-mediated angiogenesis, for a given interstitial flow $u < u_L$, capillaries grow more prominently with the flow as N_b increases. Therefore, the initial amount of matrix-bound VEGF affects the function $A_G = A_G(u)$ in the same way as the MMPs production rate: u_A and u_L increase as N_b increases, while $A_{G,m}$ increases until reaching a maximum value.

Two-parent-vessel configuration: Influence of the parent vessel separation

We have considered the two-parent-vessel configuration analyzed in the main text (see Fig. 3 in the main text) to study the influence of the parent vessel separation (d_{cap}). We have run simulations modifying d_{cap} only. As done in the main text, we consider no-flow and flow-mediated angiogenesis. For each of these cases, we consider one initial TEC in the left parent vessel and tip cells in both parent vessels. The final state of the simulations corresponding to $d_{\mathrm{cap}} = 267.5\,\mu\mathrm{m}$ and $d_{\mathrm{cap}} = 535\,\mu\mathrm{m}$ are plotted in Fig. S3, left and right column, respectively. Note that Fig. 3 in the main text corresponds to $d_{\mathrm{cap}} = 356.66\,\mu\mathrm{m}$. The upper half of Fig. S3 corresponds to $u = 0\,\mu\mathrm{m\,s}^{-1}$ while the bottom half corresponds to $u = 0.3\,\mu\mathrm{m\,s}^{-1}$. The first and third rows show the one-initial-TEC case, while the second and forth rows display the two-initial-TECs case. The overall behavior of the two-parent-vessel system does not depend on d_{cap}. The cleaved VEGF released by the upstream tip cells reaches the downstream parent vessel and triggers preferential capillary growth against the flow; see third and fourth rows in Fig. S3, where new capillaries grow against the flow in the downstream parent vessel.

Figure S3 shows how d_{cap} controls the time at which capillary growth against the flow begins. In the flow-mediated angiogenesis cases (third and fourth rows in Fig. S3), the length of the capillaries growing against the flow in the downstream parent vessel is greater for smaller d_{cap} (compare left and right columns). This means that capillaries in the downstream parent vessel start to grow earlier as d_{cap} decreases. d_{cap} also plays a minor role in the no-flow scenario. In the case of a single initial TEC (first row in Fig. S3), angiogenesis in the right parent vessel starts earlier when d_{cap} is smaller (compare left and right

columns). The results suggest that intercapillary distance may represent a mechanism to control angiogenesis initiation.

Two-parent-vessel configuration: Influence of MMPs secretion rate and matrix-bound VEGF initial distribution

We have analyzed the impact of the MMPs secretion rate on the two-parent vessel configuration (see Fig. 3 in the main text). We ran simulations in which, compared to the simulations shown in Fig. 3 in the main text, we only modify the MMPs secretion rate (β_m). In Fig. S4, left column, we plotted the final state of flow-mediated angiogenesis ($u = 0.3\,\mu\mathrm{m\,s}^{-1}$) corresponding to low β_m ($\beta_m = 0.1\,\mathrm{s}^{-1}$) for one initial TEC (top row) and two initial TECs (bottom row). We have not plotted the no-flow scenario because it is analogous to the single-parent-vessel case (see Fig. S1, left column). The one-initial-TEC case (top row, left column in Fig. S4) constitutes an unreal scenario. The initial tip cell is secreting MMPs at lower rates, which induces a low concentration of cleaved VEGF near the initial TEC. Thus, the initial tip cell does not become motile and the upstream parent vessel does not display capillary growth. However, the cleaved VEGF concentration at the downstream parent vessel reaches values high enough to trigger capillary growth against the flow. The two-initial-TECs case (bottom row, left column in Fig. S4) represents other singular scenario. Contrary to the one-initial-TEC case, the initial tip cell located in the upstream parent vessel becomes motile. The reason is the random initial distribution of the matrix-bound VEGF clusters, which is different for each simulation. In the two-initial-TECs case, there is a higher accumulation of matrix-bound VEGF clusters near the upstream initial TEC. That slight variation in the matrix-bound VEGF concentration causes the activation of the upstream initial tip cell. For the same reason (random distribution of matrix-bound VEGF), the downstream initial TEC does not become motile until the cleaved VEGF released by the upstream tip cell reaches the vicinity of the downstream parent vessel. If we compare the length of the capillaries created by the two initial tip cells, we observe that the downstream capillary is shorter, which means that the downstream initial TEC became motile later than the upstream initial TEC. The low MMPs secretion rate simulations show that flow can increase the concentration of soluble VEGF at certain locations and, hence, locally enhance vascular growth. Simulations corresponding to high MMPs secretion rates are not shown because their behavior is analogous to the high initial matrix-bound VEGF case (see Fig. S4, right column), which is described below.

We have also analyzed the impact of the initial distribution of matrix-bound VEGF on the two-parent-vessel configuration. We ran simulations in which we modified the initial amount of matrix-bound VEGF (N_b) only, by changing the number and the radius of the matrix-bound VEGF clusters. We denote $N_b^\star$ as the standard initial amount of matrix-bound VEGF considered in the two-parent-vessel problem (see, e.g., Fig. 3 in the main text). We run simulations with $N_b < N_b^\star$ and $N_b > N_b^\star$. We found that the influence of N_b is equivalent to the influence of β_m. Thus, the conclusions in the case of low N_b are analogous to the conclusions presented in the previous paragraph (see Fig. S4, left column). We plotted the final state of flow-mediated angiogenesis with $N_b = 1.37 N_b^\star$ in Fig. S4, right column. The top row corresponds to the single-initial-TEC case, while the bottom row corresponds to the two-initial-TECs case. For a given fluid velocity u, the number of new capillaries growing with the flow increases as N_b increases (compare right column, third and fourth rows in Fig. 3 in the main text with right column, first and second rows in Fig. S4). The number of new capillaries growing against the flow remains approximately constant as N_b transitions from intermediate to high values. The results show that higher initial amounts of matrix-bound VEGF improve the effectiveness of angiogenesis with the flow.

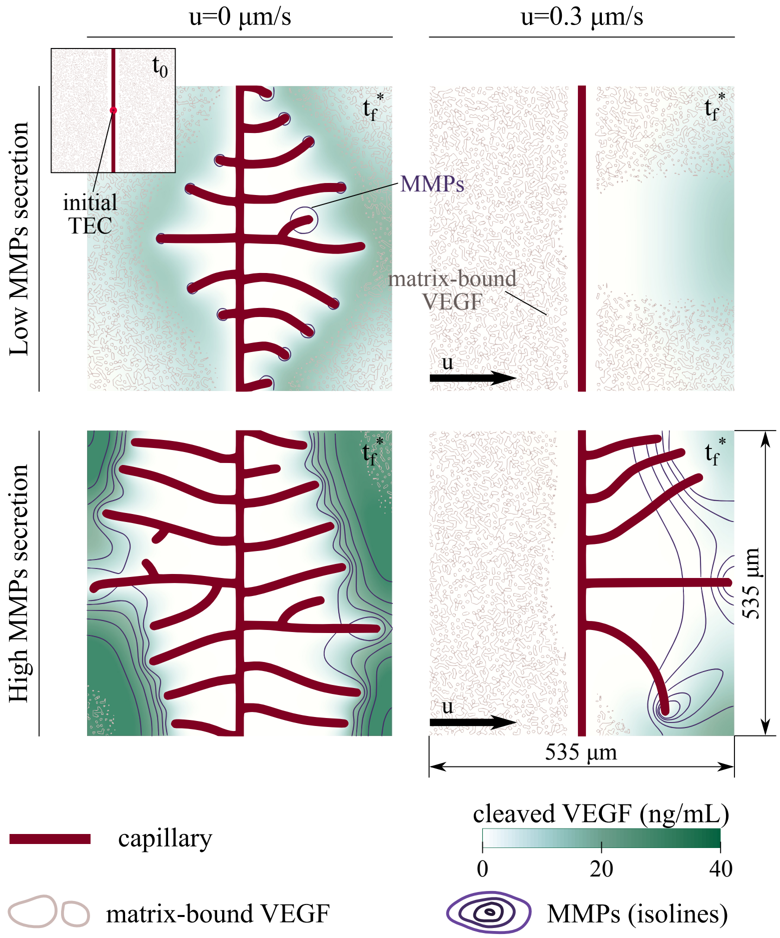

Figure S1. Influence of MMPs secretion rate in flow-mediated angiogenesis controlled by matrix-bound chemokines. Final time of the simulations corresponding to low (top row, $\beta_m = 0.03\,\mathrm{s}^{-1}$) and high (bottom row, $\beta_m = 1.65\,\mathrm{s}^{-1}$) MMPs secretion rate without (left column, $u = 0\,\mu\mathrm{m\,s}^{-1}$) and with (right column, $u = 0.3\,\mu\mathrm{m\,s}^{-1}$) interstitial flow. The snapshots show the vascular network (red), cleaved-VEGF distribution (green), MMPs isolines (purple), and contour lines of the matrix-bound VEGF clusters (gray). The flow is uniform from left to right. The inset shows the initial configuration, which includes the matrix-bound VEGF clusters, the parent vessel, and a non-motile TEC secreting MMPs. The initial TEC starts moving once all the conditions for TEC activation are achieved at the TEC's center. Note that the final time ($t_f^* \sim 1404\,\mathrm{s}$) is slightly different for each simulation.

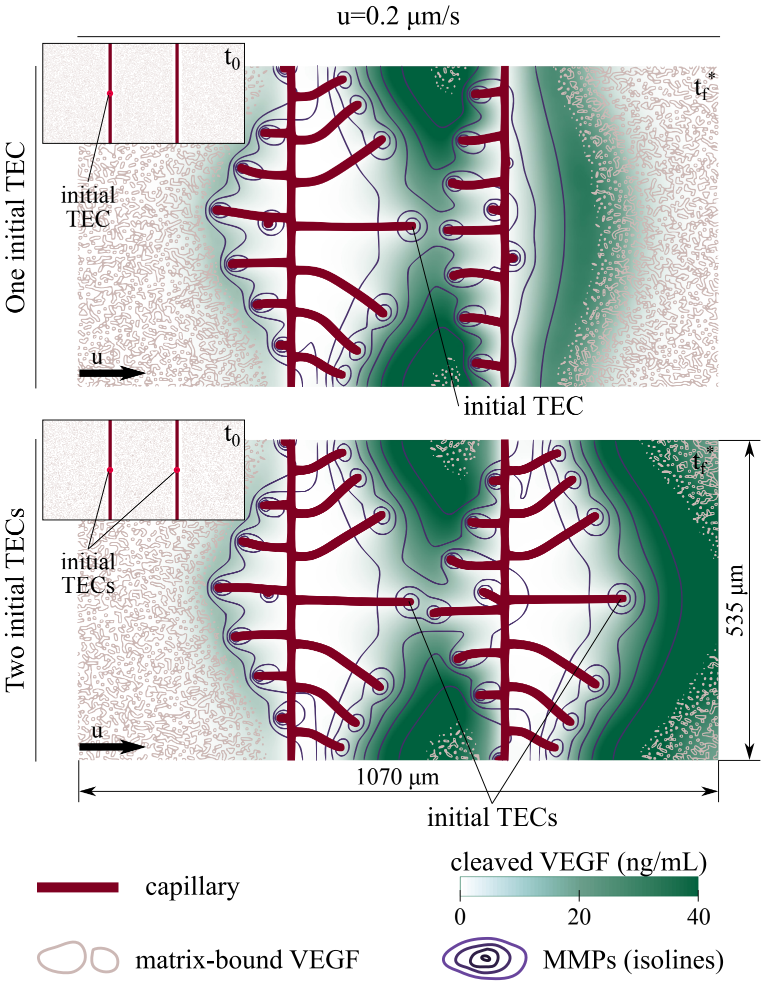

Figure S2. Angiogenesis controlled by matrix-bound chemokines in a two-parent vessel configuration with fluid velocity $u = 0.2\,\mu\mathrm{m\,s^{-1}}$. Final time of the simulations with one initial TEC in the left parent vessel (top) and tip cells in both parent vessels (bottom). The solid-line insets show the two initial configurations. The plots show the capillary network (red), cleaved-VEGF distribution (green), MMPs isolines (purple), and contour lines of the matrix-bound VEGF clusters (gray). The flow is uniform from left to right. Note that the final time ($t_f^* \sim 1170\,\mathrm{s}$) is slightly different for each simulation.

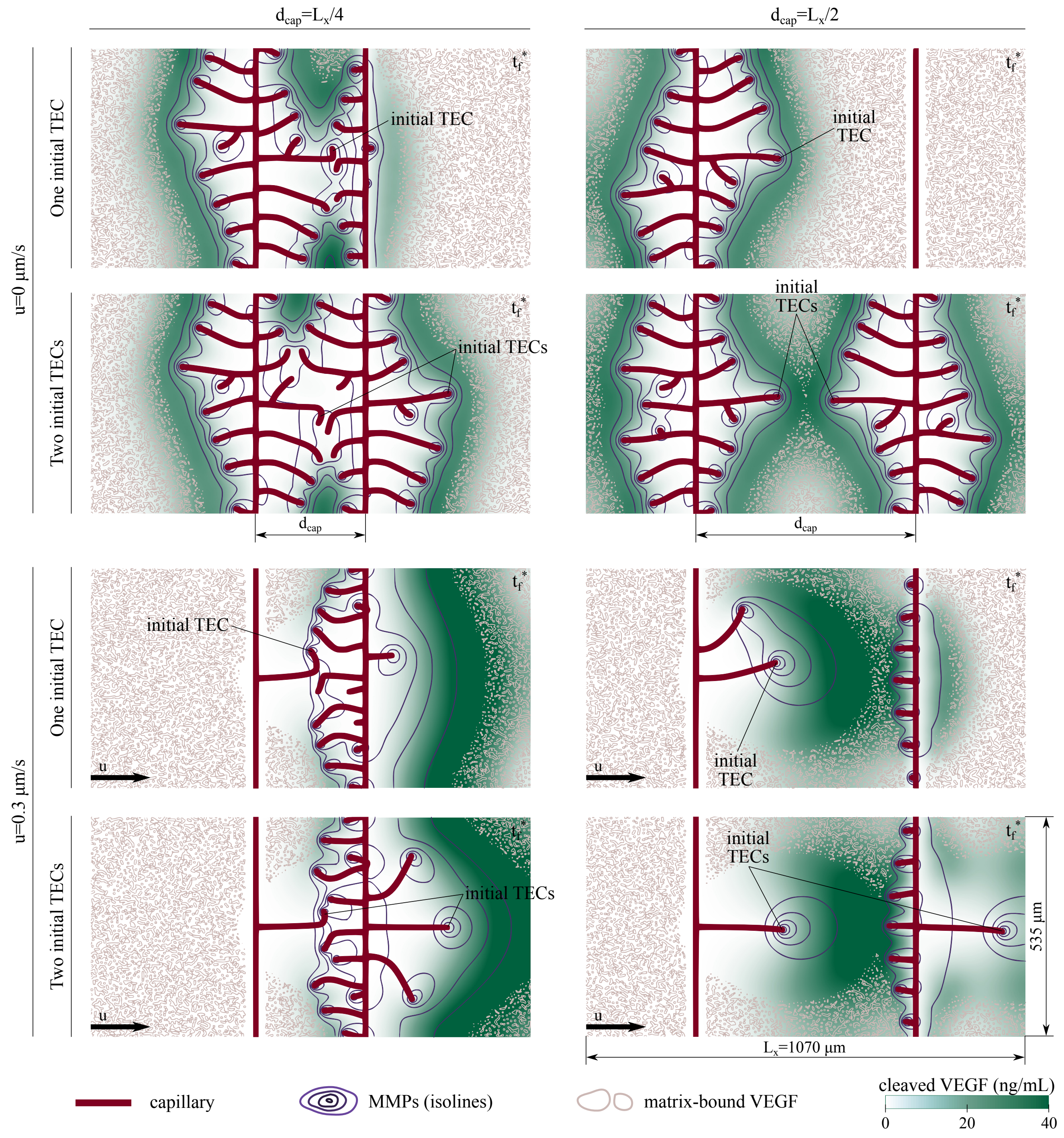

Figure S3. Influence of the parent vessel separation in flow-mediated angiogenesis controlled by matrix-bound chemokines. Final time of the simulations corresponding to parent vessel separation $d_{\text{cap}} = 267.5\,\mu\text{m}$ (left column) and $d_{\text{cap}} = 535\,\mu\text{m}$ (right column). No-flow (upper half) and flow-mediated ($u = 0.3\,\mu\text{m}\,\text{s}^{-1}$, bottom half) angiogenesis for initial conditions with one tip cell in the left parent vessel (first and third rows) and tip cells in both parent vessels (second and fourth rows). The snapshots show the vascular network (red), cleaved-VEGF distribution (green), MMPs isolines (purple), and matrix-bound VEGF contour lines (gray). The flow is uniform from left to right. The initial configurations may be found in the insets of Fig. S2. Note that the final time ($t_f^* \sim 1170\,\text{s}$) is slightly different for each simulation.

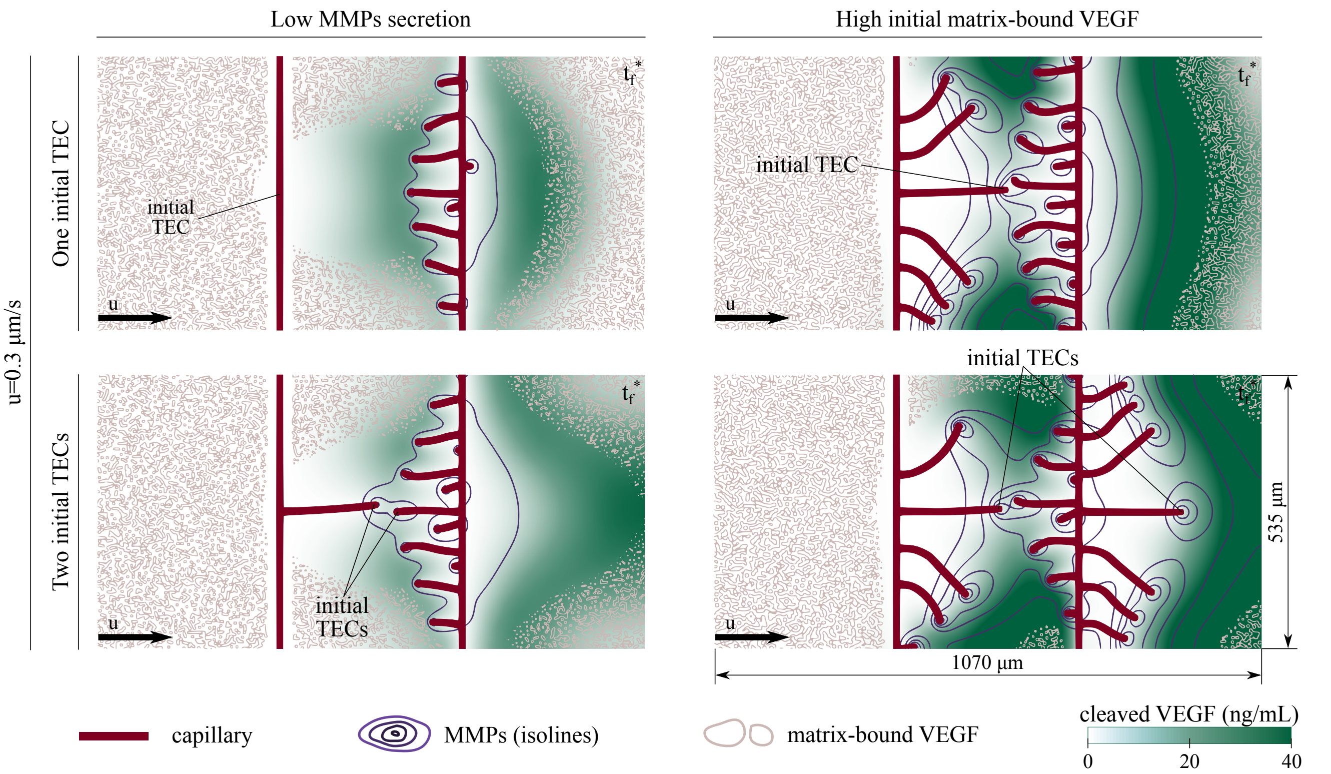

Figure S4. Influence of MMPs secretion rate and matrix-bound VEGF initial distribution in the two-parent-vessel configuration. Final time of the flow-mediated angiogenesis simulations ($u = 0.3\,\mu\mathrm{m\,s^{-1}}$) corresponding to low MMPs secretion rate (left column, $\beta_m = 0.1\,\mathrm{s^{-1}}$) and high initial amount of matrix-bound VEGF (right column, $N_b = 1.37N_b^\star$) for initial configurations with one tip cell in the left parent vessel (top row) and tip cells in both parent vessels (bottom row). $N_b^\star$ is the initial amount of matrix-bound VEGF considered in the rest of the two-parent-vessel simulations (see, e.g., Fig. S2). The initial configurations may be found in the insets of Fig. S2. The snapshots show the vascular network (red), cleaved-VEGF distribution (green), MMPs isolines (purple), and matrix-bound VEGF contour lines (gray). The flow is uniform from left to right. Note that the final time ($t_f^* \sim 1170\,\mathrm{s}$) is slightly different for each simulation.

Symbol	Description	Value	Source
M	Phase-field mobility	$1.002 \times 10^{-3}\,\mu m^2\,s^{-1}$	[3]
λ	Phase-field interfacial length scale	$1.25\,\mu m$	[3]
β	Proliferation rate	$4.987 \times 10^{6}\,mL\,ng^{-1}\,s^{-1}$	[3]
$V_{c,p}$	VEGF limit for high proliferation	$54\,ng\,mL^{-1}$	[3]
D_m	MMPs diffusion coefficient	$5\,\mu m^2\,s^{-1}$	[8]
β_m	MMPs secretion rate	$0.3\,s^{-1}$	[9]
m_{ECM}	Maximum MMPs concentration	$180\,ng\,mL^{-1}$	[9]
$U_{m,d}$	MMPs decay rate	$4 \times 10^{-5}\,s^{-1}$	[13]
$U_{m,u}$	MMPs uptake rate	$4 \times 10^{-4}\,s^{-1}$	Estimated
β_c	VEGF cleavage rate	$7.6 \times 10^{-4}\,mL\,ng^{-1}\,s^{-1}$	[8]
D_c	Cleaved-VEGF diffusion coefficient	$10.02\,\mu m^2\,s^{-1}$	[3]
$U_{c,d}$	Cleaved-VEGF decay rate	$6.41 \times 10^{-5}\,s^{-1}$	[3]
$U_{c,u}$	Cleaved-VEGF uptake rate	$0.4008\,s^{-1}$	[3]
R_{TEC}	TEC radius	$5\,\mu m$	[3]
c_{act}	Capillary condition for TEC activation	0.9	[3]
$V_{c,\mathrm{act}}$	Cleaved-VEGF threshold for TEC activation	$0.36\,ng\,mL^{-1}$	[3, 14]
δ_4	Delta-Notch distance for TEC activation	$80\,\mu m$	[3]
χ	Chemotactic velocity	$0.194\,\mu m\,s^{-1}$	[3]

Table 1. **List of model parameters**

References

1. Vilanova, G., Colominas, I. & Gomez, H. Capillary networks in tumor angiogenesis: From discrete endothelial cells to phase-field averaged descriptions via isogeometric analysis. *Int. journal for numerical methods biomedical engineering* **29**, 1015–1037 (2013).
2. Vilanova, G., Colominas, I. & Gomez, H. Coupling of discrete random walks and continuous modeling for three-dimensional tumor-induced angiogenesis. *Comput. Mech.* **53**, 449–464 (2014).
3. Vilanova, G., Burés, M., Colominas, I. & Gomez, H. Computational modelling suggests complex interactions between interstitial flow and tumour angiogenesis. *J. The Royal Soc. Interface* **15**, 20180415 (2018).
4. Gomez, H. & van der Zee, K. G. Computational phase-field modeling. *Encycl. Comput. Mech. Second. Ed.* 1–35 (2018).
5. Gomez, H., Bures, M. & Moure, A. A review on computational modelling of phase-transition problems. *Philos. Transactions Royal Soc. A* **377**, 20180203 (2019).
6. Cahn, J. W. & Hilliard, J. E. Free energy of a nonuniform system. i. interfacial free energy. *The J. chemical physics* **28**, 258–267 (1958).
7. Hellström, M. *et al.* Dll4 signalling through notch1 regulates formation of tip cells during angiogenesis. *Nature* **445**, 776–780 (2007).
8. Milde, F., Bergdorf, M. & Koumoutsakos, P. A hybrid model for three-dimensional simulations of sprouting angiogenesis. *Biophys. journal* **95**, 3146–3160 (2008).
9. Vempati, P., Mac Gabhann, F. & Popel, A. S. Quantifying the proteolytic release of extracellular matrix-sequestered vegf with a computational model. *PloS one* **5**, e11860 (2010).
10. Hughes, T. J., Cottrell, J. A. & Bazilevs, Y. Isogeometric analysis: Cad, finite elements, nurbs, exact geometry and mesh refinement. *Comput. methods applied mechanics engineering* **194**, 4135–4195 (2005).
11. Chung, J. & Hulbert, G. A time integration algorithm for structural dynamics with improved numerical dissipation: the generalized-α method. *J. applied mechanics* **60**, 371–375 (1993).
12. Jansen, K. E., Whiting, C. H. & Hulbert, G. M. A generalized-α method for integrating the filtered navier–stokes equations with a stabilized finite element method. *Comput. methods applied mechanics engineering* **190**, 305–319 (2000).
13. Urbach, C. *et al.* Combinatorial screening identifies novel promiscuous matrix metalloproteinase activities that lead to inhibition of the therapeutic target il-13. *Chem. & Biol.* **22**, 1442–1452 (2015).
14. Xu, J., Vilanova, G. & Gomez, H. Phase-field model of vascular tumor growth: Three-dimensional geometry of the vascular network and integration with imaging data. *Comput. Methods Appl. Mech. Eng.* **359**, 112648 (2020).
